# Frame-Based Stereotactic Biopsy of Canine Brain Masses: Technique and Clinical Results in 26 Cases

**DOI:** 10.3389/fvets.2015.00020

**Published:** 2015-07-27

**Authors:** John Henry Rossmeisl, Rudy T. Andriani, Thomas E. Cecere, Kevin Lahmers, Tanya LeRoith, Kurt L. Zimmerman, Denise Gibo, Waldemar Debinski

**Affiliations:** ^1^Veterinary and Comparative Neuro-Oncology Laboratory, Department of Small Animal Clinical Sciences, Virginia-Maryland College of Veterinary Medicine, Virginia Tech, Blacksburg, VA, USA; ^2^Department of Mechanical Engineering, Virginia Tech-Wake Forest School of Biomedical Engineering, Virginia Tech, Blacksburg, VA, USA; ^3^Comprehensive Cancer Center, Brain Tumor Center of Excellence, School of Medicine, Wake Forest University, Winston-Salem, NC, USA; ^4^Department of Biomedical Sciences and Pathobiology, Virginia-Maryland College of Veterinary Medicine, Virginia Tech, Blacksburg, VA, USA

**Keywords:** brain tumor, dog, glioma, neurooncology, neurosurgery

## Abstract

This report describes the methodology, diagnostic yield, and adverse events (AE) associated with frame-based stereotactic brain biopsies (FBSB) obtained from 26 dogs with solitary forebrain lesions. Medical records were reviewed from dogs that underwent FBSB using two stereotactic headframes designed for use in small animals and compatible with computed tomographic (CT) and magnetic resonance (MR) imaging. Stereotactic plans were generated from MR and CT images using commercial software, and FBSB performed both with (14/26) and without intraoperative image guidance. Records were reviewed for diagnostic yield, defined as the proportion of biopsies producing a specific neuropathological diagnosis, AE associated with FBSB, and risk factors for the development of AE. Postprocedural AE were evaluated in 19/26 dogs that did not proceed to a therapeutic intervention immediately following biopsy. Biopsy targets included intra-axial telencephalic masses (24/26), one intra-axial diencephalic mass, and one extra-axial parasellar mass. The median target volume was 1.99 cm^3^. No differences in patient, lesion, or outcome variables were observed between the two headframe systems used or between FBSB performed with or without intraoperative CT guidance. The diagnostic yield of FBSB was 94.6%. Needle placement error was a significant risk factor associated with procurement of non-diagnostic biopsy specimens. Gliomas were diagnosed in 24/26 dogs, and meningioma and granulomatous meningoencephalitis in 1 dog each. AE directly related to FBSB were observed in a total of 7/26 (27%) of dogs. Biopsy-associated clinical morbidity, manifesting as seizures and transient neurological deterioration, occurred in 3/19 (16%) of dogs. The case fatality rate was 5.2% (1/19 dogs), with death attributable to intracranial hemorrhage. FBSB using the described apparatus was relatively safe and effective at providing neuropathological diagnoses in dogs with focal forebrain lesions.

## Introduction

Over a century ago, Horsley and Clarke first described the use of a device and method for stereotactic intracranial surgery in animals (1). Since then, the field of stereotactic neurosurgery has been evolving nearly continuously and in parallel with advancements in neuroimaging technology. This has resulted in the development of numerous stereotactic devices and techniques for use in human diagnostic and therapeutic intracranial interventions, but brain biopsy remains one of the most commonly performed stereotactic neurosurgical procedures (2).

Stereotactic apparatus designed for clinical use were originally frame based, in which the patient is immobilized in a rigid headframe. The external headframe provides a stereotactic coordinate system and allows registration and correspondence of this system to the three-dimensional map of the brain generated by computed tomographic (CT) or magnetic resonance (MR) images obtained from the patient while in the headframe. Frame-based stereotactic brain biopsy (FBSB) procedures have been utilized routinely for a variety of indications in humans for nearly 50 years, and most reports indicate that FBSB is safe and effective, with procedural morbidity ranging between 0 and 13%, accuracy to within 2 mm, and diagnostic yields typically exceeding 90% (2–4). In contemporary human neurosurgical practice, frameless image-guided neuronavigational (FLIGN) biopsy techniques have been gaining popularity at many institutions, as FLIGN is the most technologically advanced, minimally invasive, and flexible technique currently available for brain biopsy. However, reports indicate that FLIGN and FBSB have similar accuracies, diagnostic yields, and overall types and rates of complications when performed by experienced neurosurgeons (5, 6).

Numerous brain biopsy techniques have also been described in dogs. These include open and minimally invasive free-hand, several FBSB techniques capable of being performed with and without image guidance using both permanent and disposable frame systems, and FLIGN procedures (7–21). Many of these investigations have focused on the description and validation of individual devices and techniques in normal canines or canine cadavers (11–13, 15–17, 19–21). Collectively, these studies demonstrate that there are multiple feasible biopsy methods capable of accurately targeting brain lesions within the range of error generally considered acceptable for clinical use in veterinary patients. However, there exist few published reports, which include a total of 98 dogs, whose specific objectives included the descriptions of the technique, clinical utility of, and complications associated with brain biopsy in cohorts of dogs with spontaneous intracranial disease (8–10, 14, 17).

The purpose of this study was to describe a technique for FBSB in a population of dogs with spontaneous forebrain mass lesions using two stereotactic headframe systems, and to report the clinical results of FBSB including diagnostic yield, intraoperative adverse events (AE), post-operative AE, and potential risk factors associated with the development of AE or non-diagnostic biopsy specimens in dogs undergoing FBSB.

## Materials and Methods

All procedures performed in this study were approved by the Virginia Tech or Wake Forest University institutional animal care and use committees under various protocols (08-048-VT, A09-143-WFU, 11-132-VT, 12-014-VT, or 14-235-VT) depending upon which therapeutic trial the dog was enrolled in, and at which institution the treatment was administered.

### Medical record review

The medical record from each dog was reviewed and the following data recorded: signalment (breed, age, sex, body weight); historical clinical signs and neurological examination findings at admission; modified canine Karnofsky performance score (KPS) at admission (22); lesion laterality (side) and anatomic location within the brain; lesion volume as determined below; date the FBSB was performed; FBSB performed with intraoperative image guidance (yes/no); duration of the FBSB procedure, as determined from the time of initial patient arrival into the CT suite for the planning scan until completion of closure of the surgical wound; final histopathological diagnosis and, where applicable, tumor grade; number of brain biopsies attempted and number obtained; and the number of non-diagnostic biopsies in each patient.

As all of the patients included in this study were enrolled in clinical trials, AE reports associated with the FBSB procedure were available in patient medical records. AE were recorded, classified, and graded according to the Cancer Therapy Evaluation Program’s CTCAE-Common Terminology Criteria for Adverse Events (23). Postprocedural AE were evaluated in only those dogs that did not proceed to a therapeutic intervention immediately following biopsy.

### Stereotactic apparatus

#### MRI Interventions Dynatech Headframe

**Figure 1:**
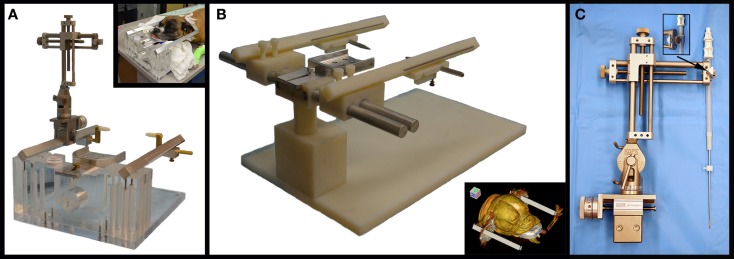
**Stereotactic instrumentation used for biopsy of canine brain masses**. **(A)** MRI Interventions Dynatech headframe with attached micromanipulator arm and canine patient in the headframe (inset). **(B)** Virginia Tech Custom headframe, with surface rendered three-dimensional reconstructed CT image of canine patient in the frame (inset). **(C)** Micromanipulator with biopsy needle and guide tube assembly mounted to the lateral arm using a knurled thumb screw coupler (inset).

This is a commercially available, MR-compatible, dedicated small animal headframe (Dynatech Machining, Union City, CA, USA) that has been used previously for stereotactic intracranial procedures in dogs (24). The frame is manufactured from plexiglass, aluminum, brass, and nylon components (Figure 1A), and is compatible with stereotactic manipulator arms outfitted for an 18.70 mm square rostrocaudal (R/C or A/P) bar. In this study, a single stereotactic micromanipulator arm was used with both headframes (1760-SB, Kopf Instruments, Tujunga, CA, USA; Figure 1A) and for both the phantom scans using needle blanks and actual FBSB procedures. This manipulator attaches to the R/C bar of the frames and can be adapted to accommodate numerous instruments, including biopsy needles. The baseplate of the manipulator is capable of calibrated movements in two planes, and can thus be used to direct instruments in oblique trajectories. The riser (vertical) and lateral arms of the manipulator contained scaled adjustment guides, which according to the manufacturer, allows placement of instruments within 0.1 mm of the desired target location. Biopsy needle guide tubes can be readily attached to the manipulator arm using a knurled thumb screw assembly that couples the guide tube seat to the peripheral aspect of the lateral arms of the micromanipulator (Figure 1C).

#### Virginia Tech Custom Headframe

The apparatus was designed by the investigators using commercial design software (Solidworks 2012, Dassault Systems, Waltham, MA, USA) and manufactured on-site (Machine Shop, College of Engineering, Virginia Tech, Blacksburg, VA, USA). Design modifications allowed for CT and MR compatibility of the device, and accommodation of a larger range of skull sizes and conformations by incorporating lateral (*x*-axis) adjustability into the rostrocaudal bar (R/C; *z*-axis) center line distance, dorsoventral (*y*-axis) adjustability through the use of a telescoping frame pedestal with a 45 cm working length, and 18.70 mm square R/C bars that accept ear bar block mounts at 10 mm increments along the length of the R/C bar from R/C bar 0 to ±50 mm (Figure 1B). The R/C bar was calibrated ±100 mm from 0. CT and MRI compatibility of the final prototype used in the study was attained by manufacturing the frame pedestal, ear bar mounts, and base plate from polyether ether ketone (PEEK, Curbell Plastics, Orchard Park, NY, USA), and R/C bars and ear bars of the frame of polyoxymethylene acetal resin (Delrin POM, DuPont, Wilmington, DE, USA). The bite plate, bite plate tongue stop screw, and *x*-plane bars were machined from aluminum sheet, bar, and rod (6AL-4V ELI, OnlineMetals, Seattle, WA, USA) stock. Stop screws and washers for use in securing the R/C bars and telescoping pedestal in fixed positions were machined from threaded and smooth Nylon 6–6 rod stock, respectively (United States Plastics Corp., Lima, OH, USA). Incremental, 1 mm, Vernier scales were laser-etched into the R/C bars, earbars, and bite plate tongue adapter (GSI Lumonics Excimer, Bedford, MA, USA) to allow for patient and stereotactic coordinate registration to the frame (25).

### Pre-operative diagnostics, imaging, and lesion volume quantification

All dogs had a diagnostic MR scan of the brain performed within 3 weeks of the biopsy procedure. These MR scans were obtained on a variety of low- and high-field (0.2–3.0 T) magnets from several referring veterinary academic and private practices using non-standardized sequences and image acquisition parameters. All pre-operative MR data sets contained pre-contrast T1- and T2-weighted images in at least two planes, at least a single planar FLAIR sequence, and post-contrast T1-weighted images in at least two planes. All dogs also had a complete blood count, serum biochemical profile, prothrombin time, and activated partial thromboplastin time performed within 24 h of the FBSB procedure.

**Figure 2:**
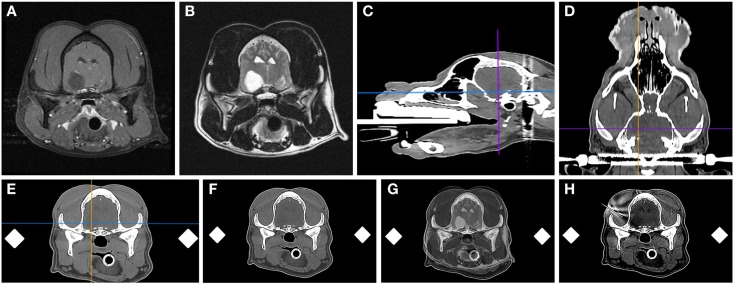
**Frame-based stereotactic biopsy planning procedure from a dog with a Grade II astrocytoma**. Diagnostic, transverse post-contrast T1-weighted **(A)** and T2-weighted **(B)** images demonstrating intra-axial, non-enhancing mass lesion in the right piriform lobe. The patient is anesthetized, immobilized in the headframe, and CT images obtained for stereotactic planning **(C–F)**, imported into image analysis software **(C–F)** and co-registered with the prior diagnostic MR **(G)**. The planned trajectory is then simulated and verified using a needle blank **(H)** and the final stereotactic coordinates recorded.

The volume of each mass lesion was determined using image analysis software (OsiriXMD, OsiriX Imaging Software, Osirix Foundation, Geneva, Switzerland). Volumes were defined from transverse T2-weighted MR images, as not all lesions demonstrated contrast enhancement (Figure 2). Manually defined regions of interest (ROIs) were generated for individual contiguous MR image slices, and volumes calculated with OsiriXMD ROI volume software, which accounts for interslice gaps.

### Stereotactic imaging and planning

Dogs were anesthetized with a total intravenous protocol consisting of premedication with a variety of benzodiazepines and fentanyl, induction with propofol, and anesthetic maintenance with propofol and fentanyl or remifentanil intravenous constant rate infusions. All dogs received cefazolin (22 mg/kg IV q 90 min) for the duration of the FBSB procedure. Following induction of general anesthesia, the dog was placed in sternal recumbency upon a rigid plexiglass backboard. The head of each dog was then affixed to the headframe using a vinylpolysiloxane putty (Express STD, 3M ESPE Dental Products, St. Paul, MN, USA) dental impression of the maxillary arcade that was molded into the biteplate. Three-point immobilization of the head occurred using the bite plate, ear bars, and memory foam and cloth pads placed ventral to the mandible, and full-patient immobilization further achieved by bolting the headframe baseplate to the plexiglass backboard using threaded nylon 6–6 bolts and nuts (MRD frame; United States Plastics Corp., Lima, OH, USA) placed through predrilled holes in the backboard or plastic C-clamps [Virginia Tech custom headframe (VTC) frame, Cavision Enterprises, Vancouver, BC, Canada]. Pre- and post-contrast (iohexol; 0.45 ml/kg, IV) CT scans were then obtained with a 16-slice scanner (Aquilion, Toshiba, Japan) using the following standardized technique: 1 mm slice thickness, with no gap, and edge enhancement algorithm with dogs in the fully instrumented headframe in sternal recumbency (12). Various image sizes and fields of view were used, depending on the size of the dog as well as the particular study the patient was enrolled in.

DICOM formatted images of the stereotactic CT and diagnostic MR were imported into image analysis software packages (OsiriXMD and Mimics v.14.1, Materialise, Leuven, Belgium), co-registered, and the stereotactic coordinates and trajectories for lesion biopsy determined by a single investigator (John Henry Rossmeisl; Figure 2). The rostrocaudal coordinates were determined based on linear (ear bar) reference markers. Mediolateral and dorsoventral coordinates and/or angular trajectories were measured directly from DICOM images using osseous anatomic landmarks, including the external sagittal crest and external surface of the skull. Generally, the needle entry point and biopsy trajectory were planned to traverse the shortest distance of normal brain between the skull and the target, and avoid sulci, major vasculature, and ventricular structures. However, in some cases in which catheter- or electrode-based therapeutic interventions were to be performed following biopsy, the biopsy trajectory deviated from these criteria such that both the diagnostic biopsy and therapeutic procedure could be performed through a single craniectomy defect (26, 27). After the trajectory was planned, the manipulator arm was attached to the headframe and the biopsy trajectory simulated using a 20-gauge needle blank (Quincke Needle, BD Medical, Franklin Lakes, NJ, USA) that was positioned at the desired needle entry point into the calvarium. The CT scan was then repeated to include the headframe and attached needle phantom using contiguous 1 mm slices using the identical field of view and magnification factors as the initial scan for each patient (Figure 2H). The target depth was measured from the external surface of the craniectomy defect to the measured position of the mid-portion of the side-cutting port of the needle within the lesion. The final stereotactic (*x*, *y*, and *z*) coordinates obtained from the phantom scan were stored in the planning software and registered to the headframe.

### Stereotactic biopsy procedure

Following stereotactic planning, the head of each dog was clipped and aseptically prepared for surgery. Dogs were transported to the operating theater while being maintained under anesthesia and immobilized in the headframe. The stereotactic coordinates were transferred from the planning station into the operating room, and then re-registered onto the frame and verified. In each dog, a routine unilateral rostrotentorial approach to the skull ipsilateral to the location of the mass was performed. Following completion of the rostrotentorial approach, a perforated drape (Apuzzo, Integra LifeSciences, Plainsboro, NJ, USA) was applied over the standard surgical drapes. The perforated drape assists mounting of the sterile manipulator arm to the non-sterile headframe. The manipulator arm and guide tube assembly was then mounted to the frame, the final stereotactic coordinates verified and recorded, and the manipulator arm rotated out of the field to allow creation of a 3- to 5-mm diameter burr-hole craniectomy defect using a high-speed pneumatic drill. The exposed dura was cauterized using bipolar cautery, and then the dura perforated with a 20-gauge needle. After creation of the burr-hole and dural defects, the biopsy needle was advanced down the guide tube to confirm that no impediments to the intended trajectory were encountered.

**Figure 3:**
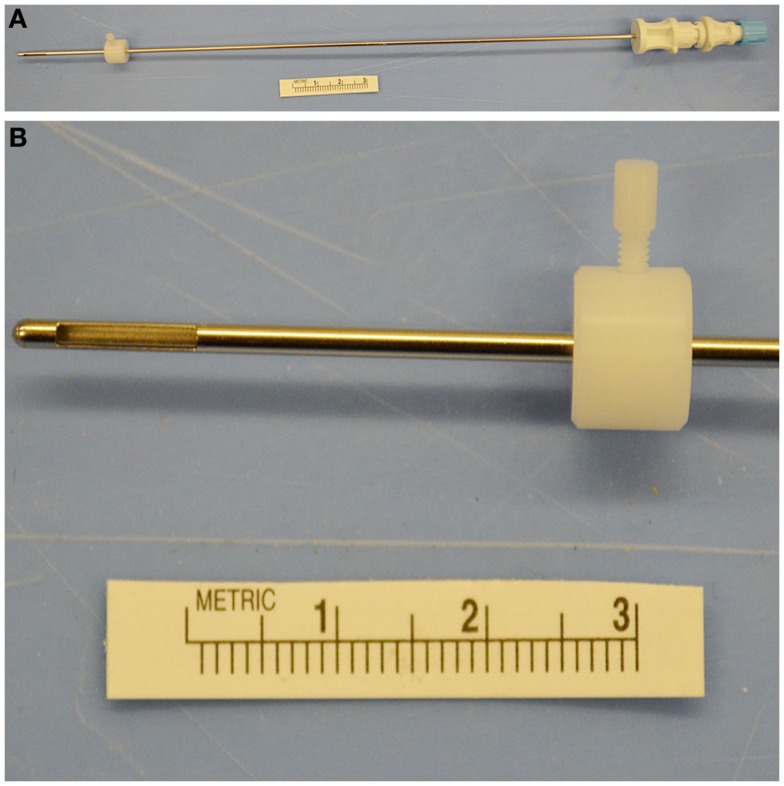
**Nashold needle with Luer lock proximal end (A) and 10 mm side-cutting channel with depth stop (A,B) used for canine brain biopsy procedures**.

A single investigator (John Henry Rossmeisl) performed all biopsy procedures using 16-gauge Nashold side-cutting aspiration biopsy needles with a 10 mm cutting channel (Integra Radionics, Burlington, MA, USA; Figure 3). The biopsy needle has a Luer lock coupler to which a syringe can be attached in order to apply slight negative pressure. When the inner cannula of the biopsy needle is rotated 180°, the side-cutting port opens. The depth to target was verified and a depth stop applied to the proximal portion of the biopsy needle.

A saline-filled syringe was attached to the needle, and the assembly flushed, and the side-cutting port closed. The needle was passed through the guide tube and gradually advanced to the planned depth using the manipulator arm controls. Once at the predetermined depth, the side-cutting port was opened by rotating the inner hub 180°, and slight negative pressure applied by withdrawing on the saline-filled syringe to pull tissue into the needle. The side-cutting port was then closed and the needle withdrawn.

**Figure 4:**
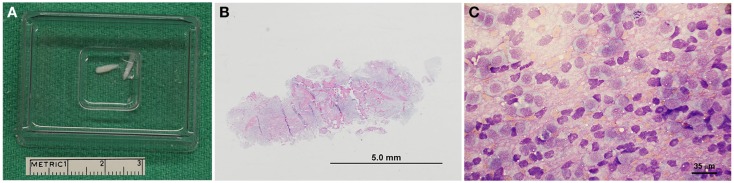
**Representative pathological features of canine stereotactic brain biopsies**. **(A)** Intraoperatively, biopsy core specimens are retrieved by flushing into a cryomold cassette. **(B)** Subgross photomicrograph of biopsy from a dog with a grade IV astrocytoma (H&E stain). **(C)** Cytologic smear preparation of an oligodendroglioma (Giemsa stain). Neoplastic cells appear to windrow within the pink proteinaceous background, and are mostly round to ovoid. Cells contain eccentrically or centrally located, round, ovoid, or reniform-shaped nuclei with stippled to coarsely stippled chromatin and a single to sometimes two small to medium-sized nucleoli. There are abundant bare nuclei. Cytoplasm is small to moderate in volume and basophilic and granular with distinct cytoplasmic borders; cytoplasm occasionally contains few to rarely moderate numbers of punctate vacuoles.

The specimen was retrieved in a similar manner, by opening the port and flushing saline through to eject the specimen into a sterile cryomold cassette (Figure 4A). Multiple specimens were typically collected by rotating the biopsy needle aperture in different directions, or alternatively by varying the target depth slightly. For instances in which the target depth was varied, one biopsy was planned that traversed the junction between normal and diseased brain tissue. Bone wax was used to occlude the burr-hole defects prior to closure. Some burr-hole defects were converted into larger craniectomy defects to accommodate implantation of guide pedestals that could accommodate passage of diagnostic-intent biopsy needles, as well as therapeutic-intent electrodes or infusion cannulae, depending on which clinical protocol the dog was enrolled in.

**Figure 5:**
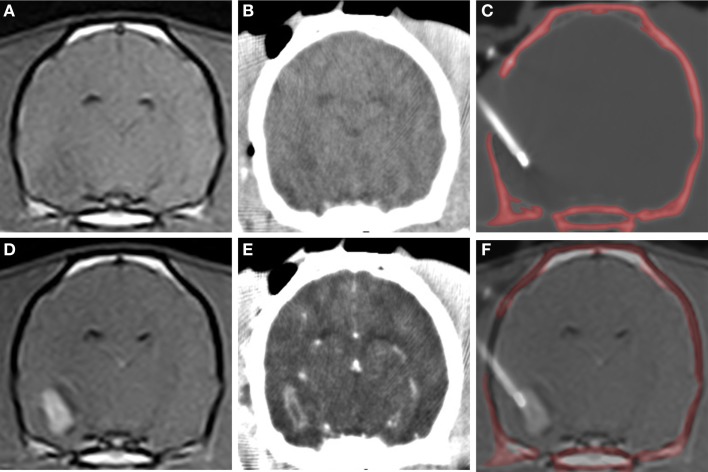
**CT-guided stereotactic biopsy procedure in a dog with a Grade III oligodendroglioma**. Transverse, pre-contrast **(A,B)** and post-contrast **(C,D)** T1-weighted MR **(A,D)** with corresponding stereotactic CT planning images **(B,E)**. Intraoperative transverse CT image **(C)** with biopsy needle *in situ*. The craniectomy defect in this patient has been enlarged to accommodate placement of an implanted guide pedestal that will later be used to introduce electrodes into the tumor for therapy. Co-registered CT and MR image **(F)** demonstrating needle placement that includes contrast enhancing lesion and the surrounding brain.

For dogs in which an intraoperative CT-guided FBSB procedure was performed, following creation of the burr-hole craniectomy defect in the operating theater, the surgical field was lavaged, covered with an impregnable, adhesive iodinated barrier drape and the patient transported under anesthesia to the CT suite while immobilized in the headframe. After positioning the patient on the CT couch, additional sterile draping was applied to the patient and CT unit, in a manner similar as described above. The manipulator arm was attached to the headframe, set to the prerecorded coordinates, and the biopsy needle positioned immediately external to the burr-hole defect, and an additional CT scan performed using the same image acquisition parameters for each patient as performed for the previously performed phantom needle scan. The biopsy trajectory was verified by co-registering the planning phantom CT with the actual biopsy CT, and then the needle advanced into the target region using the manipulator arm, and needle placement confirmed with an additional series of 1 mm contiguous CT slices through the region of the mass lesion (Figure 5), and the biopsy harvested as described above. The therapeutic protocol that each individual dog was enrolled in was the determinant of whether or not the procedure was performed with or without intraoperative image guidance. Following completion of CT-guided FBSB procedures, patients were then transported back to the operating theater, where the surgical wound was lavaged copiously and closed routinely. In all dogs, a final CT scan was performed after wound closure to evaluate for biopsy-related hemorrhage.

In those dogs in which intraoperative CT-guided FBSB was performed, the needle placement accuracy was quantified using a previously described Pythagorean technique (8). Briefly, the stereotactic coordinates of the needle tip in three planes were compared to the proposed coordinates recorded from the planning scan and the error determined using the following formula:

$$\text{Error}=\surd \left[{\left(\Delta x\right)}^{\text{2}}+{\left(\Delta y\right)}^{\text{2}}+{\left(\Delta z\right)}^{\text{2}}\right]$$

where Δ*x*, Δ*y*, and Δ*z* represent the distances from the actual needle tip to the planned target site in each of the three references axes.

All dogs received buprenorphine (0.03 mg/kg, IV or SC, q 6–8 h) for at least 36 h following recovery from the procedure. For the purposes of this study, diagnostic yield was defined as the proportion of biopsies producing a specific neuropathological diagnosis, and was calculated by dividing the number of biopsies producing diagnoses, by the total number of biopsies submitted. A failed FBSB procedure was defined as an individual case in which none of the biopsies obtained yielded a specific neuropathological diagnosis, or those cases in which brain tissue was unable to be obtained from the intended target (28).

### Processing of biopsy specimens

Biopsy specimens were fixed for 24 h in 10% neutral buffered formalin then paraffin embedded, and 5 μm thick sections were routinely stained with hematoxylin and eosin (H&E; Figure 4B) and murine monoclonal antibody against glial fibrillary acidic protein (M0761, clone 6F2, 1:150 dilution; Dako, Carpinteria, CA, USA). Biopsies were reviewed by at least two board-certified veterinary pathologists, including the pathologist that originally generated the pathology report included in the medical record, and for the purposes of this study, another pathologist blinded to the original diagnosis. In the event of diagnostic discordance between pathologists, a third blinded pathologist reviewed the biopsy, with the final diagnosis representing the majority opinion among the three pathologists. All neoplastic lesions were classified and graded according to World Health Organization criteria (29). In select cases in which multiple biopsies were obtained, one biopsy specimen was sharply divided perpendicular to the axis of the needle trajectory immediately after harvesting and submitted for ancillary cell culture, immunohistochemistry, immunoblotting, cytogenetic, or intraoperative cytologic examinations [Ref. (30); Figure 4C].

### Statistical analyses

Normal probability plots showed that age, body weight, KPS, lesion volume, number of biopsies attempted, number of biopsies obtained, target depth, mean needle error, and procedure duration were skewed. Subsequently, all continuous variables were summarized as medians (range). Categorical variables including frame type, breed, sex, lesion side, lesion location, diagnosis, tumor grade, non-diagnostic biopsies, image-guided biopsies, and presence of AE were summarized using contingency tables. Associations between frame type and perioperative characteristics were tested using a chi-square (number of procedures), Wilcoxon rank sum test (age, body weight, KPS, lesion volume, number of biopsies attempted, number of biopsies obtained, mean needle error, and target depth) and Fisher’s exact test (sex, lesion side, lesion location, diagnosis, tumor grade, image-guided biopsy, non-diagnostic biopsies, and AE). Associations between development of AE and selected perioperative characteristics were tested using Wilcoxon rank sum test (dog sequence as function of surgeon experience, KPS lesion volume, number of biopsies attempted, needle error, and procedural time) and Fisher’s exact test (lesion side, lesion location, tumor grade, and production of a non-diagnostic biopsy). Associations between producing a non-diagnostic biopsy and perioperative characteristics were tested using Wilcoxon rank sum test (dog sequence as surgeon experience, lesion volume, needle error, and procedural time) and Fisher’s exact test (lesion side, lesion location, and tumor grade). Regression analysis was used to test the association between target depth and needle error and between surgeon experience and procedural time. Association between procedural time and image-guided versus non-guided procedures was tested using the Wilcoxon rank sum test. Statistical significance was set to α = 0.05. All analyses were performed using commercial statistical software (SAS version 9.4, Cary, NC, USA).

## Results

### Patient characteristics and clinical signs

The study population consisted of 26 consecutive dogs that underwent FBSB (Table 1). All dogs had clinical signs of forebrain disease. Signs were focal in 19/26 dogs and multifocal or diffuse in 7/26. Seizures were the most common clinical sign, being observed in 22/26 cases. Interictal neurological deficits consistent with the anatomic location of the tumor were present in 20/22 of the dogs with seizures. Brachycephalic breeds (Boston terriers, Boxers, English Bulldog) comprised 12/26 cases. The median age of dogs was 8 years (range, 5–12 years) and the median body weight was 18.5 kg (range, 7–37 kg). There were 13 neutered males, 1 intact male, and 12 spayed females. The median admission KPS was 70 (range, 30–90). All dogs with structural epilepsy were receiving oral anticonvulsant medications at the time of admission, including phenobarbital (*n* = 16), levetiracetam (*n* = 7), zonisamide (*n* = 5), gabapentin (*n* = 1), and potassium bromide (*n* = 1), or various combinations of these medications (*n* = 9). All dogs in the study were also receiving oral prednisone therapy (median 0.85 mg/kg/day, range 0.33–1.4 mg/kg/day) at the time of admission. Coagulation profiles were normal in all 26 dogs. Platelet counts were within reference ranges in 24/26 dogs. In 2/26 dogs, platelet clumping prevented accurate counting, but in both of these cases platelet counts were estimated at ≥150,000/μl.

**Table 1:** **Signalments, headframe type, and lesion characteristics of dogs undergoing stereotactic brain biopsy**.

Dog No.	Breed	Age (years)	Sex	Body weight (kg)	Frame type	Lesion side/location	Diagnosis/WHO grade
1	Mixed	12	FS	11	VTC^a^	L-Parieto-occipital	Mixed glioma-III
2	Boston Terrier	8	MN	12	VTC^a^	R-Temporal-piriform	Oligodendroglioma-III
3	Boxer	7	FS	24	MRD	L-Piriform	Astrocytoma-III
4	Labrador Ret	9	MN	35	VTC^a^	R-Fronto-olfactory	Astrocytoma-IV
5	Boxer	9	MN	31	MRD	L-Piriform	Astrocytoma-II
6	Min Schnauzer	8	FS	7	VTC^a^	R-Fronto-parietal	Astrocytoma-III
7	Boston Terrier	10	MN	10	MRD	L-Piriform	Oligodendroglioma-II
8	Golden Ret	12	FS	29	MRD	R-Temporal-piriform	Astrocytoma-II
9	Am Staff Terrier	12	MN	36	VTC^a^	R-Fronto-parietal	Astrocytoma-IV
10	Boston Terrier	10	FS	11	VTC^a^	R-Piriform	Astrocytoma-II
11	Boston Terrier	11	MN	9	VTC^a^	L-Frontal	Oligodendroglioma-II
12	French Bulldog	6	FS	8	MRD	L-Parieto-occipital	GME
13	Bull Terrier	8	MN	32	MRD	R-Hemispheric	Astrocytoma-III
14	Beagle	9	FS	12	MRD^a^	R-Temporal	Astrocytoma-IV
15	Mixed	8	MN	14	MRD^a^	L-Fronto-olfactory	Astrocytoma-II
16	Am Staff Terrier	7	FS	26	MRD^a^	L-Thalamic	Astrocytoma-III
17	Labrador Ret	6	MN	31	VTC	L-Fronto-parietal	Astrocytoma-III/IV
18	Mixed	9	MN	7	MRD	R-Parieto-occipital	Oligodendroglioma-III
19	Boston Terrier	8	MN	14	MRD^a^	L-Frontal	Oligodendroglioma-II
20	Mixed	8	FS	20	MRD^a^	L-Temporal	Astrocytoma-IV
21	Mixed	7	M	17	VTC^a^	R-Fronto-olfactory	Oligodendroglioma-II
22	English Bulldog	8	FS	21	MRD^a^	L-Frontal	Astrocytoma-II
23	Boston Terrier	11	MN	10	VTC	R-Temporal	Astrocytoma-IV
24	Boxer	12	FS	26	VTC	R-Parasellar	Meningioma-I
25	Boxer	8	FS	37	MRD	R-Temporal-piriform	Oligodendroglioma-III
26	Mixed	5	MN	27	MRD	R-Fronto-parietal	Astrocytoma-IV

*^a^Biopsy performed with intraoperative CT-guidance*.

*GME, granulomatous meningoencephalitis; FS, female spayed; L, left; M, male; MN, male neutered; MRD, MRI Instruments Dynatech headframe; R, right; VTC, Virginia Tech Custom headframe*.

### MR lesion features and FBSB techniques

All dogs had solitary forebrain lesions identified on pre-operative MR examinations. Fourteen lesions were located in the right forebrain, and 12 in the left forebrain (Table 1). Lesions were classified as intra-axial in 25/26 dogs, and extra-axial in the remaining dog. Nineteen lesions were described as well marginated, and the remaining seven poorly demarcated from the surrounding neuropil. Some degree of contrast enhancement was observed in 20/26 lesions, although the patterns and intensities of contrast enhancement were highly variable. The median lesion volume was 1.99 cm^3^ (range, 0.55–6.48 cm^3^). FBSB was performed with the VTC headframe in 11 cases, and with the MRD headframe in 15 cases. CT-guided FBSB procedures were performed in 14/26 dogs, and the 12 remaining biopsies were completed without intraoperative image guidance (Table 1). There were no significant differences between surgeon experience (dog order in which FBSB procedure was performed), patient age, sex, bodyweight, KPS, lesion laterality, lesion location, lesion volume, number of biopsies attempted, number of biopsies obtained, number of non-diagnostic biopsies, overall rate of AE, final diagnosis, tumor grade, number of procedures, number of image-guided procedures, or duration of the biopsy procedure for FBSB performed with the VTC or the MRD headframe (Tables 1 and 2). The duration of CT-guided FBSB procedures (median 138 min, range 117–191 min) was significantly longer than FBSB procedures performed without intraoperative image guidance (median 100 min, range 84–118 min; *p* < 0.001). There were no significant associations observed between surgeon experience and procedural time (slope = −0.74; 95% CI = −2.35 to 0.88; *t*(24) = −0.94; *Y* = 134.36–0.74(X); *r*^2^ = 0.04; *p* = 0.36), or needle error with target depth (slope = −0.01; 95% CI = −0.03–0.02; *t*(12) = −0.76; *Y* = 1.94 – 0.01(X); *r*^2^ = 0.05; *p* = 0.46).

**Table 2:** **Summary of continuous patient, lesion, and biopsy variables by headframe type**.

Frame total No. dogs (CT-guided biopsies)	Age (years)	Body weight (kg)	Admission KPS	Lesion volume (cm^3^)	Biopsies attempted	Biopsies obtained	Needle error (mm)	Target depth (mm)
MRD *n* = 15 (6)	10 (5–12)	20 (7–35)	70 (40–80)	2.22 (0.55–6.48)	2 (1–4)	2 (1–4)	1.55 (1.1–3.4)	26 (14–44)
VTC *n* = 11 (8)	8 (6–12)	12 (10–37)	70 (30–90)	1.27 (0.69–4.98)	2 (1–3)	2 (1–3)	1.50 (0.9–2.0)	21 (15–66)
*p* Value	0.11	0.94	0.77	0.14	0.12	0.06	0.84	0.35

*All data presented as medians and ranges*.

*KPS, Karnofsky performance score*.

### Biopsy characteristics

In the 26 dogs, a total of 58 biopsies were attempted and 56 biopsy samples obtained and submitted for histopathological analysis. The median number of biopsies attempted per dog was 2 (range, 1–4), and no individual dog experienced a failed FBSB procedure. Twenty-five FBSB procedures were performed using a single needle trajectory. Neuroepithelial neoplasms accounted for 24/26 diagnoses, including astrocytomas in 16/26 dogs, oligodendrogliomas in 7/26 dogs, and a mixed glioma in 1 dog. A meningioma was diagnosed in the dog with the extra-axial mass lesion, and granulomatous meningoencephalitis was diagnosed in one dog (Table 1). The overall diagnostic yield was 94.6%, with 53/56 individual biopsies producing specific neuropathological diagnoses. Necrosis, hemorrhage, and astrogliosis, (*n* = 1), extensive necrosis precluding cytoarchitectural evaluation (*n* = 1), and inclusion of only normal brain tissue in the sample (*n* = 1) accounted for the three non-diagnostic biopsies. Among the variables examined for associations with the risk of obtaining a non-diagnostic biopsy, only needle error was significant (Tables 3 and 4).

**Table 3:** **Analysis of continuous risk factors associated with obtaining a non-diagnostic stereotactic brain biopsy**.

Risk factor	Non-diagnostic biopsies		Diagnostic biopsies		*p* Value
	*N*	Median (range)	*N*	Median (range)	
Surgeon experience (procedure number)	3	10.0 (2.0–20.0)	23	14.0 (1.0–26.0)	0.53
Lesion volume (cm^3^)	3	1.0 (0.9–1.4)	23	2.2 (0.6–6.5)	0.12
Needle error (mm)	2	2.9 (2.3–3.4)	12	1.5 (0.9–2.1)	0.05*
Procedural duration (min)	3	118 (86–191)	23	140 (84–149)	0.87

*Statistically significant (*p* ≤ 0.05).

**Table 4:** **Analysis of categorical risk factors associated with obtaining a non-diagnostic stereotactic brain biopsy**.

Risk factor	Category	Number	Non-diagnostic biopsies	Diagnostic biopsies	*p* Value
			*N* (%)	*N* (%)	
Lesion side	Left	12	1 (8.3)	11 (91.7)	1.00
	Right	14	2 (14.3)	12 (85.7)	
Lesion location	Fronto-olfactory	3	0 (0.0)	3 (100.0)	0.91
	Frontal	3	0 (0.0)	3 (100.0)	
	Frontoparietal	4	0 (0.0)	4 (100.0)	
	Piriform	4	1 (25.0)	3 (75.0)	
	Temporal-piriform	3	1 (33.3)	2 (66.7)	
	Temporal	3	1 (33.3)	2 (66.7)	
	Parieto-occipital	3	0 (0.0)	3 (100.0)	
	Parasellar	1	0 (0.0)	1 (100.0)	
	Thalamic	1	0 (0.0)	1 (100.0)	
	Hemispheric	1	0 (0.0)	1 (100.0)	
WHO tumor grade	I	1	0 (0.0)	1 (100.0)	1.00
	II	9	1 (11.1)	8 (88.9)	
	III	8	1 (12.5)	7 (87.5)	
	IV	7	1 (14.3)	6 (85.7)	
CT-guided biopsy	No	12	1 (8.3)	11 (91.7)	1.00
	Yes	14	2 (14.3)	12 (85.7)	

*CT, computed tomographic; WHO, World Health Organization*.

### Adverse events

Overall, AE attributable to the FBSB procedure were observed in 7/26 (27%) of dogs. Seven dogs proceeded immediately to invasive neurosurgical interventions following FBSB, including one dog that experienced an FBSB-associated intraoperative AE. Thus, data from 19/26 dogs were included in assessment of FBSB-related post-operative AE. Intraoperative AE were observed in 4/7 dogs, and post-operative AE in 3/7. The case fatality rate for dogs undergoing FBSB was 5.2% (1/19). Intraoperative AE included intracranial or intratumoral hemorrhage from the biopsy track [*n* = 2; Grade V (fatal) in one dog and clinical effects unable to be evaluated in one dog], Grade I cerebrospinal fluid (CSF) leakage from iatrogenic penetration of the lateral ventricle with the biopsy needle (*n* = 1), and a Grade I break in aseptic technique in the CT suite (*n* = 1). Aspiration of blood and CSF from the biopsy needle accounted for the two biopsy attempts in which brain tissue was unsuccessfully obtained. The case in which lateral ventricular penetration occurred required that the biopsy be performed using a second trajectory. The one dog in which post-operative effects of FBSB-related intracranial hemorrhage could not be evaluated was discharged from the hospital 3 days after receiving the therapeutic intervention. The case in which a break in aseptic technique occurred was treated with a four-week course of cefovecin (8 mg/kg SC q 14 days). No adverse post-operative clinical effects were observed in either the dog in which the lateral ventricle was penetrated or in the dog in which there was a break in aseptic technique.

Post-operative AE included seizures in two dogs (Grade I, *n* = 1 and Grade II, *n* = 1) and transient, Grade II exacerbation of pre-existing neurological deficits (depression in consciousness, hemiparesis, and circling) in one dog. Both dogs that experienced post-operative seizures had been diagnosed with structural epilepsy prior to the FBSB, were receiving anticonvulsant therapy prior to biopsy, and received intravenous diazepam as a single or intermittent bolus injection to treat post-operative seizures. The dog that experienced a worsening of clinical signs of unilateral forebrain signs was treated with mannitol (1.0 g/kg, IV) and prednisone (1 mg/kg/day PO), and returned to its baseline neurological status 4 days after recovering from FBSB. No significant risk factors associated with the development of adverse effects were identified (Tables 5 and 6).

**Table 5:** **Analysis of continuous risk factors for the development of adverse events associated with stereotactic brain biopsy**.

Risk factor	Adverse events present		Adverse events absent		*p* Value
	*N*	Median (range)	*N*	Median (range)	
Surgeon experience (procedure number)	7	15 (6–25)	12	18.5 (5–26)	0.59
Lesion volume (cm^3^)	7	2.5 (1.4 – 6.5)	12	1.7 (0.7 – 3.5)	0.17
Number biopsies attempted	7	2 (1–4)	12	2 (1–4)	0.60
Needle error (mm)	4	1.6 (1.4–2.0)	4	1.8 (1.3–2.1)	0.78
Admission KPS	7	70 (50–90)	12	80 (40–90)	0.55

*KPS, Karnofsky performance score*.

**Table 6:** **Analysis of potential risk factors for the development of adverse events associated with stereotactic brain biopsy**.

Risk factor	Category	Number	AE present [*N*; (%)]	AE absent [*N*; (%)]	*p* Value
Lesion side	Left	9	4 (44.4)	5 (55.6)	0.65
	Right	10	3 (30.0)	7 (70.0)	
Lesion location	Fronto-olfactory	2	1 (50.0)	1 (50.0)	0.74
	Frontal	2	1 (50.0)	1 (50.0)	
	Frontoparietal	3	1 (33.3)	2 (66.7)	
	Piriform	2	0 (0.0)	2 (100.0)	
	Temporal-piriform	2	1 (50.0)	1 (50.0)	
	Temporal	3	0 (0.0)	3 (100.0)	
	Parieto-occipital	2	1 (50.0)	1 (50.0)	
	Parasellar	1	0 (0.0)	1 (100.0)	
	Thalamic	1	1 (100.0)	0 (0.0)	
	Hemispheric	1	1 (100.0)	0 (0.0)	
Lesion type	Astrocytoma	13	4 (30.8)	9 (69.2)	0.69
	Oligodendroglioma	5	2 (40.0)	3 (60.0)	
	GME	1	1 (100.0)	0 (0.0)	
WHO tumor grade	I	1	0 (0.0)	1 (100.0)	0.06
	II	7	2 (28.6)	5 (71.4)	
	III	5	4 (80.0)	1 (20.0)	
	IV	5	0 (0.0)	5 (100.0)	

*AE, adverse event; WHO, World Health Organization*.

**Figure 6:**
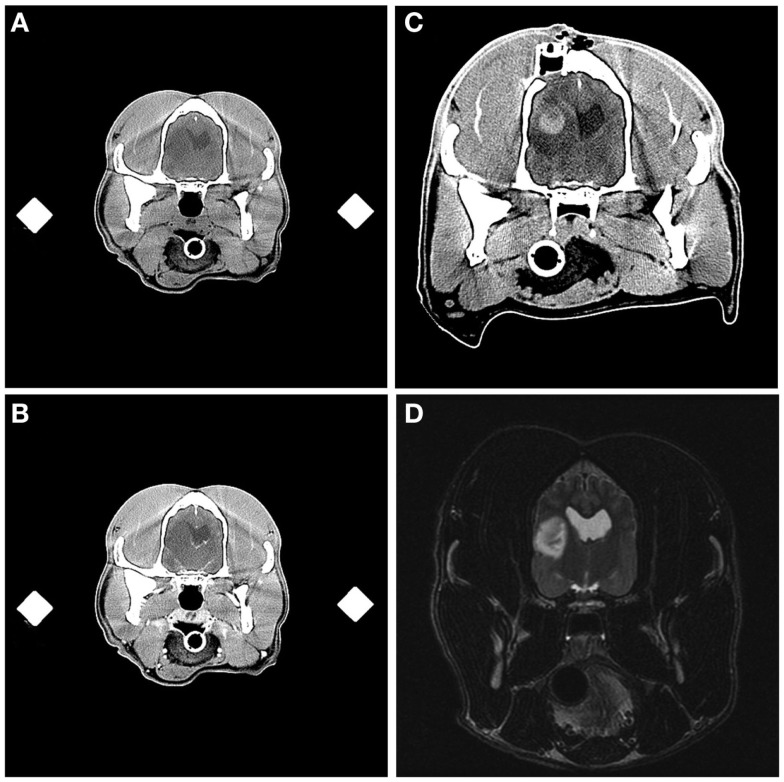
**Intracranial hemorrhage resulting from frame-based stereotactic biopsy of a Grade III oligodendroglioma**. Transverse, pre- **(A)** and post-contrast **(B)** stereotactic planning CT scans demonstrating hypoattenuating and non-enhancing mass lesion in the temporal region. On the immediate post-biopsy, pre-contrast CT scan, biopsy-induced intracranial hemorrhage is apparent as an ovoid, hyperattenuating lesion contained within the dorsal and central aspect of the previously described hypoattenuating mass **(C)**. The craniectomy and implanted catheter guide pedestal **(C)** through which the biopsy was performed are visible in the parietal bone dorsal to the lesion. Initial diagnostic T2-weighted, transverse MR image **(D)**, illustrating the hyperintense lesion in the temporal lobe of this patient.

Aside from the changes anticipated from the FBSB procedure, including the presence of craniectomy defects and small amounts of gas or hemorrhage in the soft-tissues overlying the calvarium, abnormalities were observed in immediate post-biopsy CT examinations in 6/26 dogs. These included intracranial or intratumoral hemorrhage (Figure 6) in two dogs, pneumocephalus contained within in the biopsy track in two dogs, ventricular pneumocephalus in the dog in which the lateral ventricle was compromised during the biopsy, and hypoattenuating mass effect in the brain parenchyma surrounding the biopsy track, consistent with edema, in the one dog that experienced transient clinical decline.

## Discussion

The described stereotactic apparatus allowed for safe and effective stereotactic brain biopsy. Based on our operational definition, the overall diagnostic yield was 94.6%, which is similar to other reports of FBSB in dogs and humans (3, 10, 14). Given the lack of significant differences observed between patient demographics, lesion characteristics, number of intraoperative image-guided procedures, proportion of non-diagnostic biopsies, and incidence dogs of AE between the MRD and VTC headframes, we demonstrate that both of these apparatus can be used successfully for FBSB in dogs with focal forebrain masses both with and without intraoperative image guidance. Although successful use of a free-hand brain biopsy technique performed with MRI-imaging planning data translated to the operating theater has been described in dogs with encephalitis, this is the first report describing the clinical utility of FBSB performed without intraoperative image guidance in dogs with spontaneous intracranial lesions (18).

Although no individual dog in this study was considered to have a failed stereotactic biopsy procedure, defined as one in which a definitive neuropathological diagnosis is not established based on the tissue obtained, 5.4% (3/56) of individual biopsies obtained in this study yielded non-specific changes that were not representative of the primary underlying pathology (28). Sampling of areas of necrosis, gliosis, or normal brain accounted for the changes observed in non-diagnostic biopsy specimens. These perilesional changes have also been shown to account for non-representative biopsy of humans with high-grade gliomas (6, 28). While the needle placement errors observed in this study were within previously reported ranges for CT-guided FBSB in dogs, needle placement error was significantly greater in non-diagnostic biopsy specimens, emphasizing the need for accurate execution of the planned trajectory when performing FBSB (9, 10, 20).

Calculation of the needle error is subject to inherent patient, operator, and instrument inaccuracies associated with overall error determinations, such as maintenance of rigid patient immobilization throughout the procedure, correct coordinate registration to the frames, trajectory planning, needle insertions, and coregistration of pre-operative and intraoperative image sets (19, 20). Additional technical factors, such as the amount of negative pressure used to pull tissue samples into the biopsy window, were neither controlled for nor quantified in this study, and could have also contributed to the generation of non-diagnostic biopsy samples. The appropriate amount of negative pressure required to obtain quality biopsy specimens is variable, and in our experience can be influenced by the type of lesion sampled. For example, we observed that the gelatinous matrix characteristic of many canine oligodendrogliomas readily lends itself to the harvesting of robust samples using side-cutting needles with application of slight negative pressure that are ideally suited for cytopathological preparations (31). However, the consistency of oligodendrogliomas also presents challenges when attempting to preserve the integrity of the tissue core during transfer from the biopsy needle. The size of mass lesions subjected to biopsy could have also have contributed favorably to our observed diagnostic yield. All masses in this cohort of dogs were >4.5 mm at their maximal two-dimensional cross sectional diameter, which is a size reportedly amenable to biopsy based on prior reports examining accuracies of frame-based CT-guided biopsy (11, 16), and we found no significant associations between lesion volume and the risk of non-diagnostic biopsy. Although we were unable to identify a statistically significant association between lesion volume and the risk of non-diagnostic biopsy, it is clinically notable that the median volume of masses yielding successful biopsies was greater than twice the median volume of mass lesions from which non-diagnostic biopsies were obtained. Thus, the potential effects of lesion volume on diagnostic yield may warrant further investigation in larger numbers of dogs subjected to FBSB.

Studies have indicated that increasing the number of biopsies harvested and usage of intraoperative histopathological examination of frozen sections and cytopathological evaluations increase the diagnostic yield, and thus, reduce the likelihood of failed stereotactic biopsy procedures (5, 31–33). While harvesting several biopsy specimens deceases the risk for failed stereotactic biopsy, some studies have indicated that increasing the number of biopsies is also associated with an increasing risk for AE, especially when multiple biopsy trajectories are used (28, 34). In this study, multiple biopsies were obtained using a single trajectory whenever possible to potentially mitigate the development of AE, but intraoperative AE required the use a second trajectory to obtain diagnostic tissue in one case. Although both intraoperative cryosections and cytological preparations were performed in selected cases reported here, they were neither used consistently nor prepared or processed uniformly, and thus, we did not evaluate their impact on diagnostic yield in this study.

Adverse events directly attributable to FBSB were observed in 7/26 (27%) of dogs, although the potential clinical sequelae of the AE could be assessed in 19/26 dogs, which included 6/7 dogs experiencing AE. Biopsy-associated clinical morbidity occurred in 3/19 (16%) of dogs, and the case fatality rate was 5.2% (1/19 dogs). The morbidity and mortality rates observed in this study are similar to previous investigations of FBSB investigations in dogs with spontaneous intracranial disease, which have reported case fatality rates of 7–8% and incidences of biopsy-associated morbidity ranging from 12 to 27% (9, 13). Intracranial hemorrhage, exacerbation of seizures, and transient neurological deterioration are recognized complications associated with brain biopsy in dogs and humans, and accounted for all of the symptomatic AE observed in this study (3, 10, 14, 18, 31).

Biopsy-induced hemorrhage is recognized as one of the most common AE encountered during FBSB, and is also the AE most often associated with procedural morbidity and mortality in both dogs and humans (4–6, 10, 14, 31, 32). Similar to humans, reports of canine stereotactic brain biopsies performed to date, suggest that the majority of symptomatic intracranial biopsy-related hemorrhages will declare themselves clinically in the acute post-operative period (10, 14, 35, 36). As has been previously demonstrated in humans and dogs, post-biopsy imaging examinations were useful for the detection of procedural hemorrhage and other complications, such as edema, and assisted with management of AE (10, 14, 35, 36). In humans undergoing stereotactic brain biopsy, asymptomatic biopsy-related hemorrhage is commonly observed on post-operative imaging examinations, with an approximate incidence of 50% (34–36). Delayed clinical deterioration secondary to hemorrhage can occur up to several days following biopsy in humans, and the risk for delayed hemorrhagic AE is considered minimal in people with negative post-biopsy imaging examinations that recover from the procedure without incident (35, 36).

Both dogs in this case series that experienced post-operative seizures had pre-existing tumor associated structural epilepsy and were receiving anticonvulsant therapy at the time of enrollment. In both of these cases, the provoked seizures were possible to control with single or intermittent boluses of diazepam. However, medically intractable seizures have been observed following FBSB in dogs (10). Two intraoperative AE in this study, including iatrogenic perforation of the lateral ventricle and a break in aseptic technique in the CT suite, did not result in any clinically apparent AE and resolved with no specific or minor specific interventions. Asymptomatic ventricular pneumocephalus was also reported in a prior study investigating the clinical utility of minimally invasive, free-hand brain biopsy in dogs with encephalitis (18).

Numerous risk factors associated with the development of clinical morbidity have been identified in humans undergoing FBSB. Some studies have independently recognized the histologic diagnosis of highly vascularized, high-grade glioma as a risk factor for morbidity, as well as the immune status of the patient, or lesions that involve the basal ganglia or thalamus (4, 6, 34). Other factors, such as patient age, pre-operative neurological status, number of biopsy specimens taken, and target lesion size, have been identified in some series and not others as factors associated with post-operative morbidity and mortality (4, 34, 37). Although we did not identify any significant risk factors associated with the development of AE in this study, we commonly performed FBSB in patients with high-grade gliomas that often involved subcortical basal nuclei. Our inability to detect risk factors may have been affected by sample size limitations. The morbidity and mortality rates observed here may also have been favorably influenced by a patient population in which predominantly telencephalic lesions were sampled, with only one dog undergoing FBSB for a thalamic lesion. Stereotactic biopsy of brainstem lesions has been associated with high mortality rates in dogs, although the specific locations of biopsy targets within the brainstem were not always specified in animals experiencing fatal complications (14).

Recognizing the inherent difficulties associated with presumptive diagnosis of intra-axial mass lesions-based exclusively on contemporary MR sequences, as well as the inability of MR imaging to reliably distinguish between different types and grades of canine gliomas, this study reinforces the importance of histopathological diagnosis for the optimal management of patients with intracranial disease (38, 39). In this cohort of dogs, one dog with a presumptively diagnosed glioma based on imaging was ultimately diagnosed with focal macrogranulomatous GME following FBSB, which significantly and favorably altered this patient’s therapy and prognosis.

Due to lack of universal access to necropsy or surgical resection specimens, as well as the potential for some of the therapies administered to alter the original phenotype of the tumor, we made no attempt to evaluate the diagnostic accuracy of FBSB (27). The diagnostic accuracy of FBSB can be defined as the proportion of biopsies that generate diagnoses that are reflective of the primary pathological process present in an individual patient, and it is determined by comparison with “gold standard” samples that are obtained following surgical resection of the lesion or at necropsy.

An additional limitation of this study is the institutional selection bias inherent in the population of dogs subjected to FBSB. With the exception of the single dog with the parasellar meningioma, dogs were selected for FBSB based upon the presence of an intra-axial forebrain mass lesion with imaging features compatible with a glioma for subsequent enrollment in investigative clinical trials for the treatment of gliomas (38). However, one of the most frequent indications for stereotactic biopsy in humans is pathological diagnosis of deep-seated intra-axial masses, such as gliomas (2, 4, 6). Thus, while our results with respect to the diagnostic yield of FBSB and risks associated with non-diagnostic biopsy and AE are relevant to dogs with focal gliomas involving the forebrain, our conclusions are not applicable to the utility of FBSB for lesions that are non-neoplastic, multifocal, or located in the caudal brainstem. However, this report represents a large cohort of dogs in which glial tumors were histopathologically diagnosed antemortem, and indicates that FBSB can be used successfully with acceptable clinical morbidity in dogs with forebrain gliomas.

Given the technique with which intraoperative image-guided procedures were performed in this study, which required moving the patient into and out of the operating theater and CT suite twice, it is not unexpected that CT-guided FBSB procedural times were significantly longer than those performed without intraoperative image guidance. Previous studies of CT-guided biopsies in dogs describe performance of a minimally invasive burr-hole approach in the radiology suite (10, 13, 14). However, we deviated from these techniques because we often simultaneously instrumented the patient for a future therapeutic-intent procedure while performing FBSB. These preparations required more extensive surgical approaches and permanent implantation of electrode or catheter guide pedestals into the skull (27). We believed that it was indicated to perform these procedures in the aseptic environment of the operating theater due to their anticipated length and invasiveness. No associations between procedural time and surgeon experience were identified in this study, and the median duration of intraoperative image-guided biopsy was 138 min. While our intraoperative image-guided procedures took longer than the 60 min required by Moissonnier to perform CT-guided FBSB (14), our procedural times were well within the ranges published in another study in dogs, which reported durations of CT-guided biopsy initially lasting 180–240 min, but eventually being reduced to 90–120 min as the surgeon gained experience (9).

In conclusion, the two headframe systems utilized in this study allowed for successful stereotactic biopsy of solitary, predominantly intra-axial forebrain masses both with and without intraoperative CT-guidance. Among the 19 dogs in which AE related to FBSB could be evaluated, 84% (16/19) recovered without clinical complications, including 2 dogs that experienced intraoperative AE. Intracranial hemorrhage, seizures, and transient neurological decline secondary to brain edema were the most common AE observed in this study and accounted for all symptomatic AE. With modifications to the micromanipulator arm, both headframes described here could be used in MR-guided FBSB, which may further improve the outcomes associated with stereotactic brain biopsy in dogs.

## Author Contributions

Study (JR, WD) and apparatus design (JR, RA), data acquisition (JR, TC, KL, TL, KZ, DG), data analysis and interpretation (JR, TC, KL, TL, KZ, DG, WD), drafting of the manuscript (JR), critical revision of the manuscript (RA, TC, KL, TL, KZ, DG, WD), and final approval and acknowledgment of accountability (JR, RA, TC, KL, TL, KZ, DG, WD).

## Conflict of Interest Statement

Dr. John Henry Rossmeisl reports grants from National Institutes of Health/National Cancer Institute, grants from Wallace Coulter Foundation, during the conduct of the study; in addition, Dr. John Henry Rossmeisl has a patent US 8,992,517 B2 Irreversible Electroporation to Treat Aberrant Cell Masses with royalties paid to Angiodynamics, Inc., a patent VTIP 10-130 Tissue Electroporation Application Delivery Devices pending, and a patent WO 2012/154284 A2-Fiber array for optical imaging and therapeutics pending. The other co-authors declare that the research was conducted in the absence of any commercial or financial relationships that could be construed as a potential conflict of interest.
